# Silent progression in patients with rheumatoid arthritis: is DAS28 remission an insufficient goal in RA? Results from the German Remission-plus cohort

**DOI:** 10.1186/s12891-017-1528-y

**Published:** 2017-04-19

**Authors:** Philipp Sewerin, Stefan Vordenbaeumen, Annika Hoyer, Ralph Brinks, Christian Buchbender, Falk Miese, Christoph Schleich, Sabine Klein, Matthias Schneider, Benedikt Ostendorf

**Affiliations:** 1 Department of Rheumatology & Hiller Research Unit, University Hospital Düsseldorf, Moorenstreet 5, Duesseldorf, 40225 Germany; 2 German Diabetes Centre, Institute for Biometry and Epidemiology, Auf’m Hennekamp 65, Duesseldorf, 40225 Germany; 3 Department of Diagnostic and Interventional Radiology, University Hospital Düsseldorf, Moorenstreet 5, Duesseldorf, 40225 Germany

**Keywords:** Magnetic resonance imaging, Rheumatoid arthritis, RAMRIS, Therapy monitoring, Remission, Silent progression

## Abstract

**Background:**

Remission is arguably the ultimate therapeutic goal in rheumatoid arthritis (RA). Applying modern strategies, clinical remission can be achieved in a substantial number of patients with early RA (ERA). Even in those patients, the number and scope of erosions can increase. We, therefore, investigated the value of MRI for the detection of radiological progression in patients with DAS28 improvement and/or clinical remission of the German Remission-plus cohort.

**Methods:**

Data-sets of 80 RA patients (according to 2010 ACR/EULAR criteria) from the Remission-plus study cohort, who fulfilled the following criteria, were retrospectively analysed: availability of two consecutive MRI scans (low-field MRI, follow-up interval 1 year) of the clinically dominant hand and wrist, and the presence of DAS28 (CRP) scores at both time points, which was used to assess disease activity.

**Results:**

Seventy-one of the 80 investigated patients presented a numerical improvement of the DAS28 (CRP) after 12 months (DAS28(CRP) T0 average (Ø) 4.96, SD 1.2; DAS28 T4 (12 month) Ø 2.6, SD 1.0), 73% of them also improved in the RAMRIS-Score, while 24% demonstrated an increase despite DAS28 improvement and 3% showed equal values. 48% of patients who improved in the DAS28 reached EULAR remission. 41% of these patients had an increase in the RAMRIS Erosion-subscore after 12 months. When considering EULAR response criteria (non-response (*n* = 7), moderate response (*n* = 19), good response (*n* = 45)), an increase of erosions was found in 71.4% of non-responders, 52.6% of moderate responders, and 31.1% of good responders after 12 months, all compared to baseline.

**Conclusion:**

Up to 40% of patients in this study demonstrated a progressive erosive disease detected by MRI despite DAS28 improvement or EULAR remission. Future studies are needed to determine the prognostic clinical impact of disease progression in MRI despite clinical remission, and to investigate if DAS28 remission may be an insufficient therapeutic goal and should be accompanied by MRI remission criteria.

## Background

Remission in rheumatoid arthritis [RA] is arguably the ultimate goal of an anti-rheumatic therapy [1, 2]. With modern therapeutic strategies, this goal can be achieved in the majority (up to 80%) of patients with early RA (ERA) [3]. In this context, remission has been defined as a “state of absent disease activity”. In contrast, flares are defined as “a substantial increase of disease activity” associated with more radiological progression and worse functional outcome [4]. Hence, continuous remission is the desired target state. A variety of response scores for RA patients based on clinical and serological data have been proposed and applied in clinical trials [5]. Among these, the American College of Rheumatology (ACR) response criteria, which rely on a relative change of five core set variables [6], and the European League Against Rheumatism (EULAR) response criteria, which are based on an absolute change of the composite Disease Activity Score in 28 joints (DAS28) including the ACR/EULAR remission criteria [7–9], are most common.

In 2002, the OMERACT (Outcome Measures in RA Clinical Trials) magnetic resonance imaging (MR)I-group introduced a highly reliable sum-score (RA MRI Score (RAMRIS)) [10] based on the semi-quantitative rating of the severity of synovitis, bone marrow edema and bone erosions in the joints of the hand and wrist [10, 11]. The RAMRIS system has been shown to be a sensitive tool for the evaluation of therapy in patients receiving conventional synthetic and biologic DMARDs (Disease-modifying anti-rheumatic drugs) [12, 13] similar to scores measuring clinical and serological parameters [14]. However, Emery et al. reported a weak correlation between the individual change of the RAMRIS and the change of the DAS28 and C-reactive protein (CRP) levels, respectively. This was thought to be due to superior sensitivity of MRI compared to DAS28 and CRP [15]. It is additionally known that the number and scope of erosions can increase instead of clinically low disease activity or remission (measured by DAS28). In particular, the existence and continuous presence of bone marrow edema as depicted by MRI is the strongest predictor for bony erosiveness in RA patients [16, 17]: Imaging studies with ultrasound and MRI revealed signs of synovitis and/or bone marrow edema in patients with clinical remission (i.e. according to ACR or EULAR criteria). This phenomenon, often denominated “silent progression”, thus came into scientific focus [18, 19]. Consequently, the question was raised whether extended remission criteria which incorporate modern imaging tools could be of superior value compared to clinical composite indices [20].

We, therefore, investigated the value of MRI for the detection of erosive changes in patients with DAS28 improvement and/or remission of the German Remssion-plus cohort [21].

## Methods

### Study design

Retrospective analysis was done on the Remission-plus cohort in which the data had been prospectively evaluated [21].

### Patients cohort

Datasets of 146 RA patients from the Remission-plus study cohort who fulfilled the ACR/EULAR 2010 Criteria for RA [21] were retrospectively analysed in this study. Moreover, 80 patients who fulfilled advanced inclusion criteria consisting of (1) availability of two consecutive MRI scans (follow-up interval 1 year) of the clinically dominant hand and wrist, (2) the presence of DAS28 (CRP) scores at both time points and (3) had an DAS28 > 3,2 at T0 were investigated.

### Clinical assessment

The following EULAR core set of variables was recorded: patient’s global assessment of overall disease activity, number of tender and swollen joints, erythrocyte sedimentation rate (ESR) and C-reactive protein (CRP (<5 mg/l)).

The DAS28 [22] was used to assess disease activity. Changes of disease activity were graded by the following classification criteria: DAS28 < 2.6 = clinical remission, ≤ 3.2 mild disease activity < 5.2 moderate disease activity and > 5.2 severe disease activity [23, 24].

### EULAR response assessment

Therapy response was graded by the following improvement criteria proposed by the EULAR committee [7, 8]: DAS28 decrease >1.2 units and endpoint score <3.2 = good response, DAS28 decrease >1.2 units and endpoint score >3.2 or DAS28 decrease 0.6–1.2 units and endpoint score <5.1 = moderate response, DAS28 decrease <0.6 or DAS28 decrease 0.6–1.2 units and endpoint score >5.1 = poor response.

### Imaging procedure [low-field MRI examination]

All examination were performed with the same low-field strength 0.2-T dedicated extremity MRI unit (Esoate, C-Scan, Esaote Biomedica Germany GmbH), and the same dedicated, dual phased-array coil. The clinically dominant hand was examined. Patients with renal dysfunction and known allergic reactions to gadolinium-diethylenetriaminepentaacetic acid (Gd-DTPA) were excluded from the study. The imaging protocol comprised pre- and post-contrast (i.v. gadolinium-based MRI contrast material, e.g. Magnevist, Schering AG, Berlin) T1-weighted images with a maximum slice thickness of 3 mm in at least two orthogonal planes and coronal fat-suppressed short tau inversion recovery (STIR) sequences [in detail coronar T1-weighted before contrast agent, coronar fat-suppressed STIR before contrast agent, 3-D GE T1-weighted after contrast agent with multiplanar reconstruction in three slide positions, coronar T1- weighted after contrast agent, axial T1-weighted after contrast agent].

### MRI-scoring (RAMRIS)

MRI images were scored in each centre by MRI trained rheumatology specialists according to the RAMRIS based on OMERACT recommendations [10]. MR images were read in consensus by two board-certified radiologists with special expertise in musculoskeletal MRI and trained for RAMRIS scoring.

### Statistical analysis

Results of the analyses were reported as absolute numbers and percentages where appropriate. Data management and analysis was performed with SAS, version 9.3 (SAS Institute, Cary, North Carolina).

## Results

### Characteristics of patients

Overall, 146 patients were included in the Remission-plus cohort. 64 patients were excluded due to pregnancy, death, movement or loss to follow-up. Finally, 80 patients were included in the final evaluation (30% male, 70% female). 19% showed a disease duration of less than 6 months, while 53% presented disease duration of less than 24 months, and 47% showed a disease duration of more than 24 months. The entry patient characteristics are outlined in Table 1.

**Table 1:** Patient’s characteristics at T0 (begin of the study) including the sex, disease duration, seropositivity, conventional x-rays, clinical and laboratory parameters and MRI scores (RAMRIS and RAMRIS-Subscores)

*N* = 80	
Male	24 [30%]
Female	56 [70%]
Disease-duration <6 month	15 [19%]
Disease-duration <24 month	42 [53%]
Disease-duration ≥24 month	38 [47%]
RF pos.	47 [59%]
CCP antibody pos.	49 [62%]
Erosiv x-rays	23 [32%] [missings *n* = 9]
CRP [mg/l]	9.35 [SD 15.61; Min 1, Max 88]
DAS28	2.98 [SD 1.2; Min 1, Max 6,8]
RAMRIS	7,78 [SD 7.16; Min 0, Max 33]
SYN-subscore	2,34 [SD 2.54; Min 0, Max 11]
ERO-subscore	4,39 [SD 4.74; Min 0, Max 19]
BME-subscore	1,05 [SD 1.96; Min 0, Max 12]

### Erosiveness at TO

At T0 (begin of the study) conventional x-rays of the hands were performed. 23 of the 71 patients (32%, 9 missings) already showed at least one erosion in plane x-rays of the hand while 48 patients had no detectable erosions. Regarding the concordant MRI scans, 44 of these 48 patients [92%] showed at least one single erosion in the MRI scans (Erosion (ERO)-subscore ≥ 1).

### Clinical improvement and MRI results

**Fig. 1:**
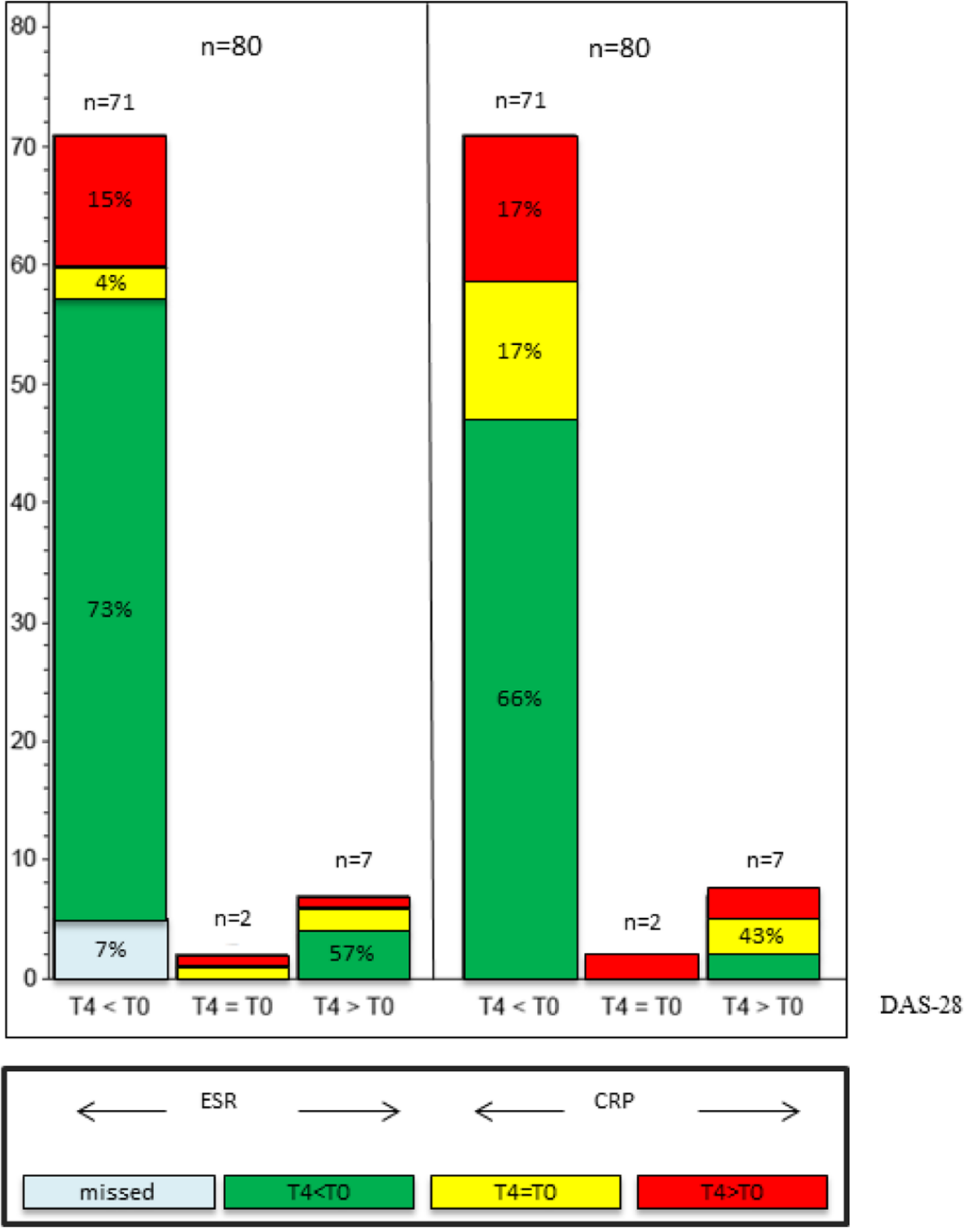
Comparison of DAS28 response to changes in ESR and CRP. Each *left column*: patients who improved in DAS28 after 12 month (T4 < T0); each *middle column*: patients with equal values (T4 = T0) and each *right column*: patients who worsened in DAS28 after 12 month (T4 > T0). *Green coloured sections*: improvement in ESR or CRP; *yellow coloured sections*: equal values; *red coloured sections*: worsening in ESR or CRP

Seventy-one of the 80 analysed patients presented a clinical improvement of the DAS28 after 12 months (T4), while two showed a stable disease activity and 7 worsened (DAS28(CRP) T0 average (Ø) 4.96; SD 1.2; DAS28 T4 (12 month) Ø 2.6; SD 1.0) (Fig. 1).

**Fig. 2:**
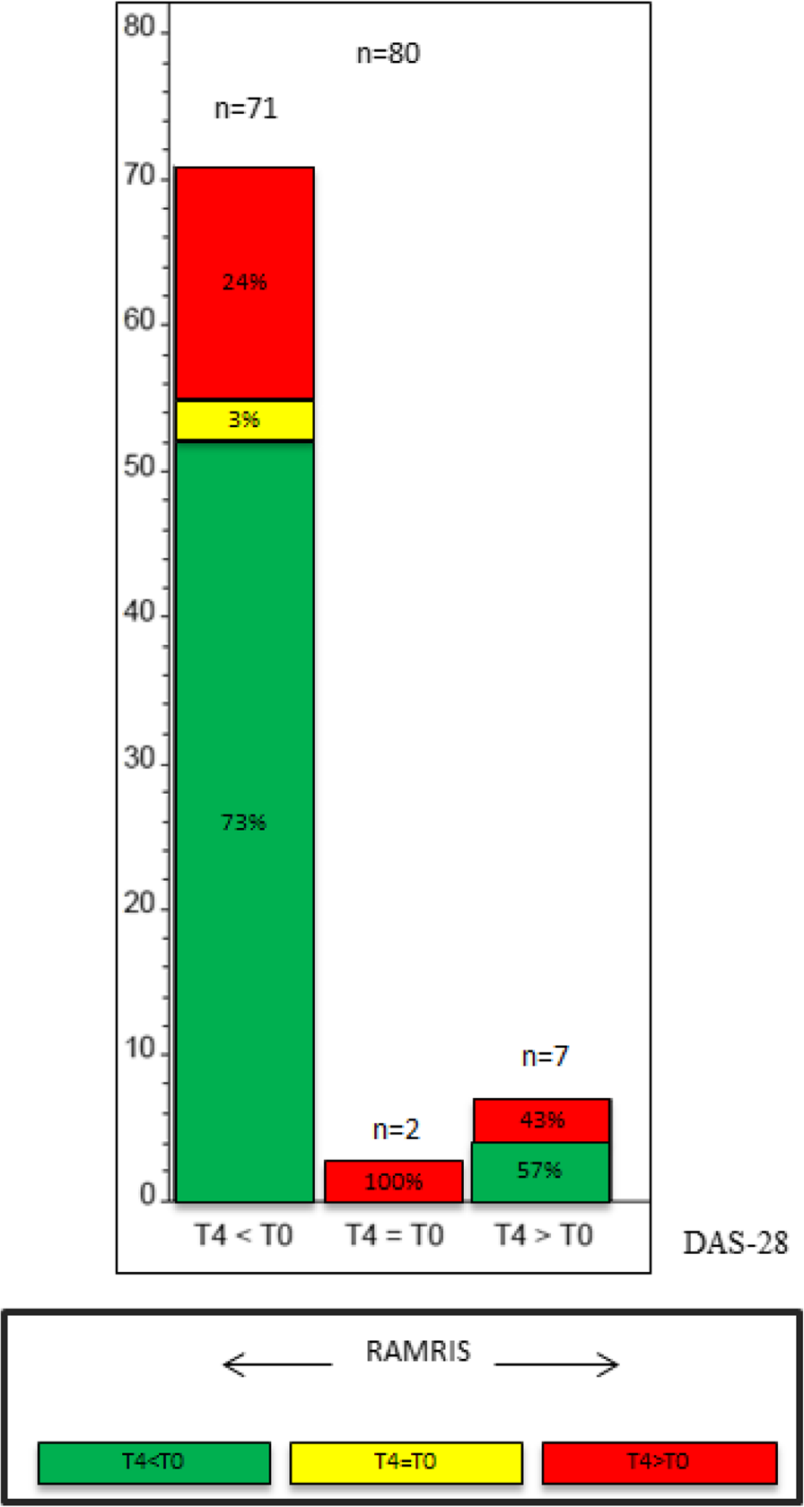
Comparison of DAS28 response to changes in RAMRIS. *Left column*: patients who improved in DAS28 after 12 month (T4 < T0); *middle column*: patients with equal values (T4 = T0); *right column*: patients who worsened in DAS28 after 12 month (T4 > T0). *Green coloured sections*: improvement in RAMRIS; *yellow coloured sections*: equal values; *red coloured sections*: worsening in RAMRIS

After 12 months, 73% of the 71 patients who improved in DAS28 showed a lower RAMRIS-Score, while 24% worsened despite DAS28 improvement, 3% showed equal values (Fig. 2). When considering RAMRIS-Subscores, 41% (*n* = 29) of these 71 patients had more erosions on MRI compared to baseline (ERO-subscore T4 > T0), while 39% showed less erosions (T4 < T0) after 12 months. Hence, 8 of 29 patients who worsened in ERO-subscore showed a difference of 1 point while 21 (approximately 72%) patients changed by at least 2 points. Regarding the affected joints, the proximal metacarpophalangeal (MCP) 2-joint was most frequently affected by worsening in ERO-subscore (9/29) followed by the trapezoid bone (6/29), the proximal MCP-3 (4/29) and proximal MCP-4 joint (4/29). Only 1/29 patients worsened in the PIP joints. We studied in addition the impact of age, sex, antibody status, systemic inflammation (CRP) and RAMRIS-subscores and found no relevant association.

**Fig. 3:**
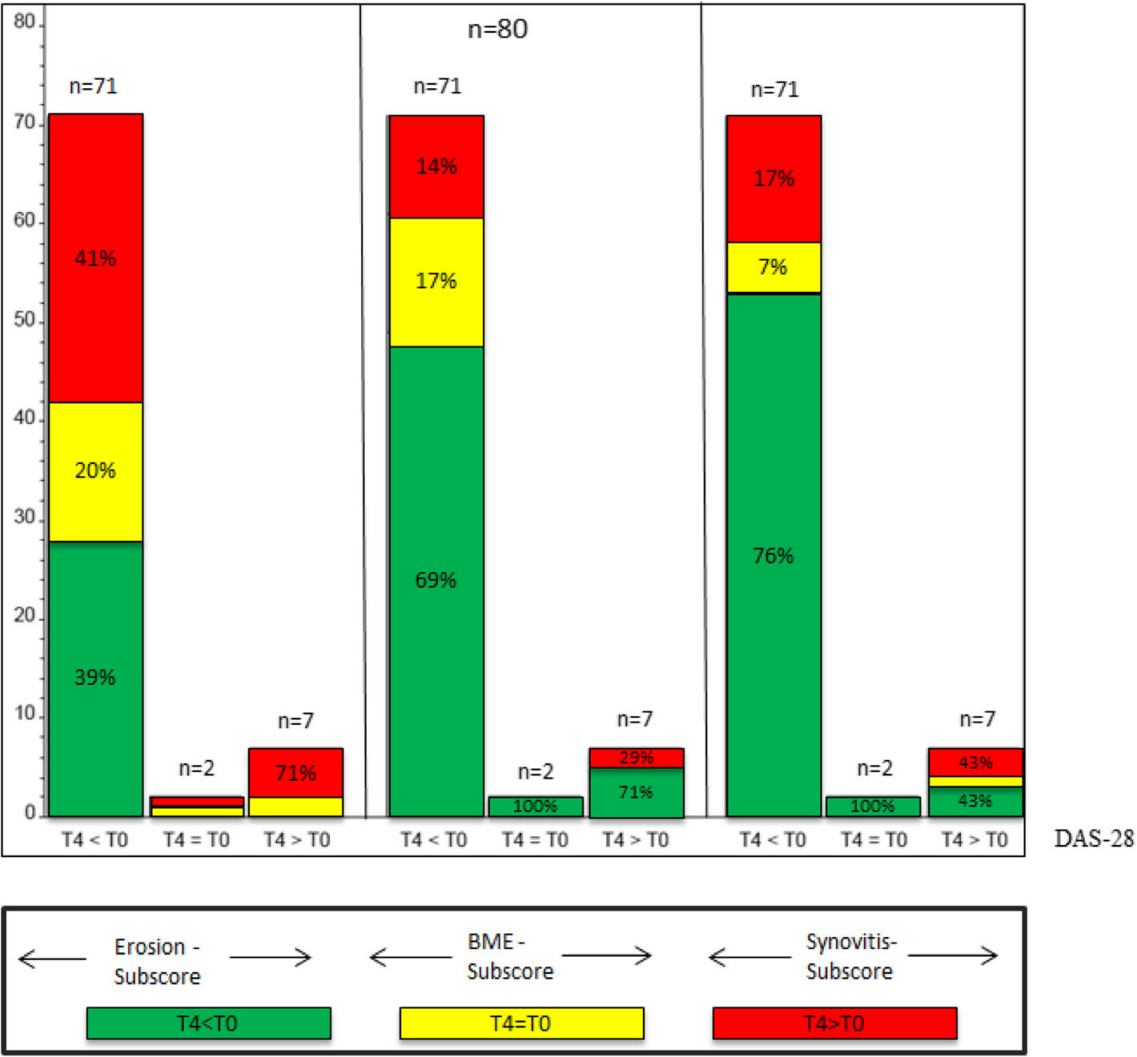
Comparison of DAS28 response to changes in Erosion-subscore, Bone-Marrow Edema (BME)-subscore and Synovitis-subscore of the RAMRIS Score. Each *left column*: patients who improved in DAS28 after 12 month (T4 < T0); each *middle column*: patients with equal values (T4 = T0); each *right column*: patients who worsened in DAS28 after 12 month (T4 > T0). *Green coloured sections*: improvement in the RAMRIS-subscores; *yellow coloured sections*: equal values; *red coloured sections*: worsening in RAMRIS-subscores

In contrast, the intensity of Bone Marrow Edema (BME) and Synovitis (SYN) in MRI decreased in accordance to clinical improvement in 69% (BME) and 76% (SYN) (Fig. 3).

**Fig. 4:**
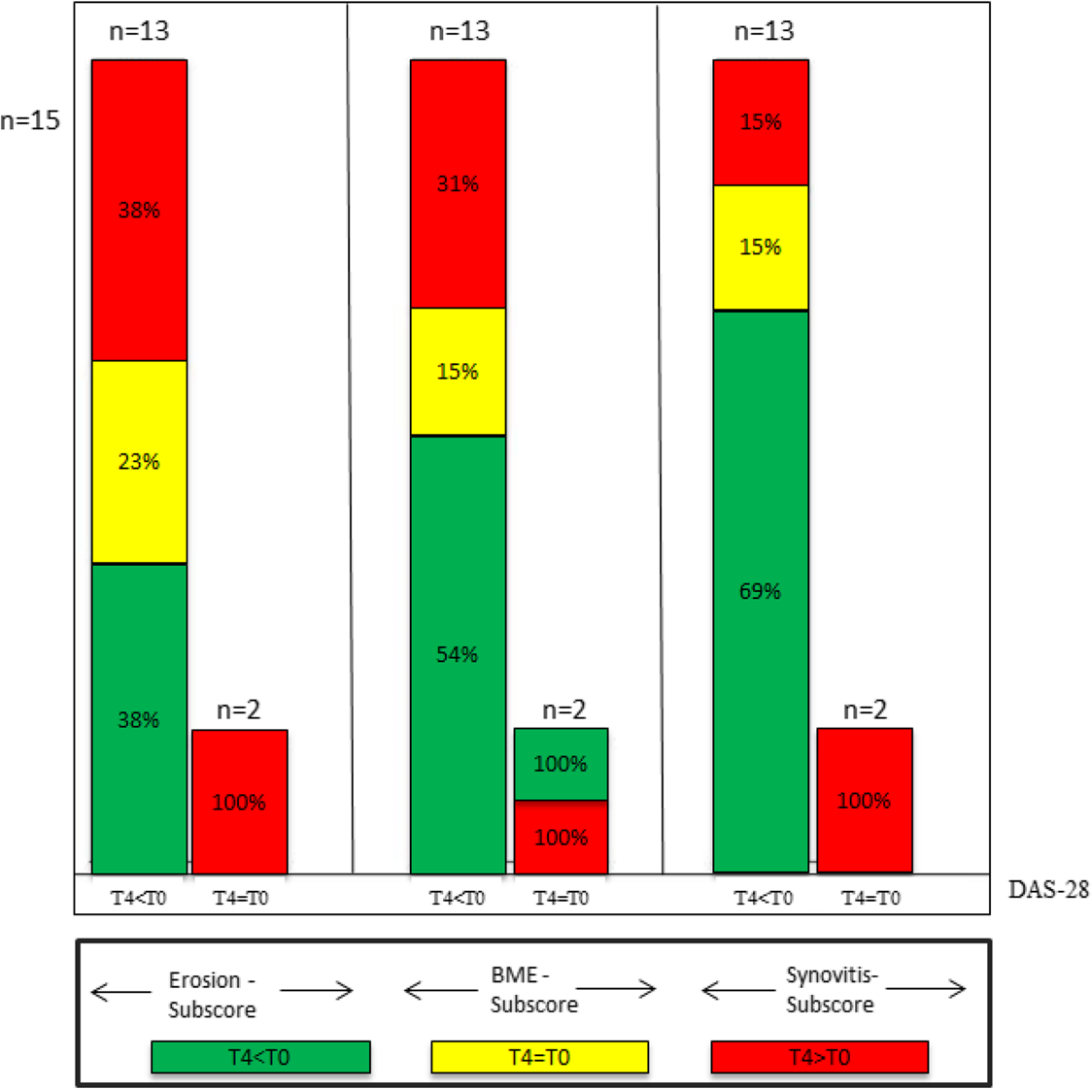
Comparison of DAS28 response to changes in Erosion-subscore, Bone-Marrow Edema (BME)-subscore and Synovitis-subscore of the RAMRIS Score of patients with short disease duration (less than six month). Each *left column*: patients who improved in DAS28 after 12 month (T4 < T0); each *right column*: patients with equal values (T4 = T0). *Green coloured sections*: improvement in the RAMRIS-subscores; *yellow coloured sections*: equal values; *red coloured sections*: worsening in RAMRIS-subscores

In a subgroup of patients with a short disease duration (<6 month, *n* = 15), tantamount results were found: 38% showed less erosions after 12 months of treatment, 23% a stable erosion score and 38% increased erosions score despite DAS28 improvement (Fig. 4).

### MRI criteria with respect to EULAR remission

**Fig. 5:**
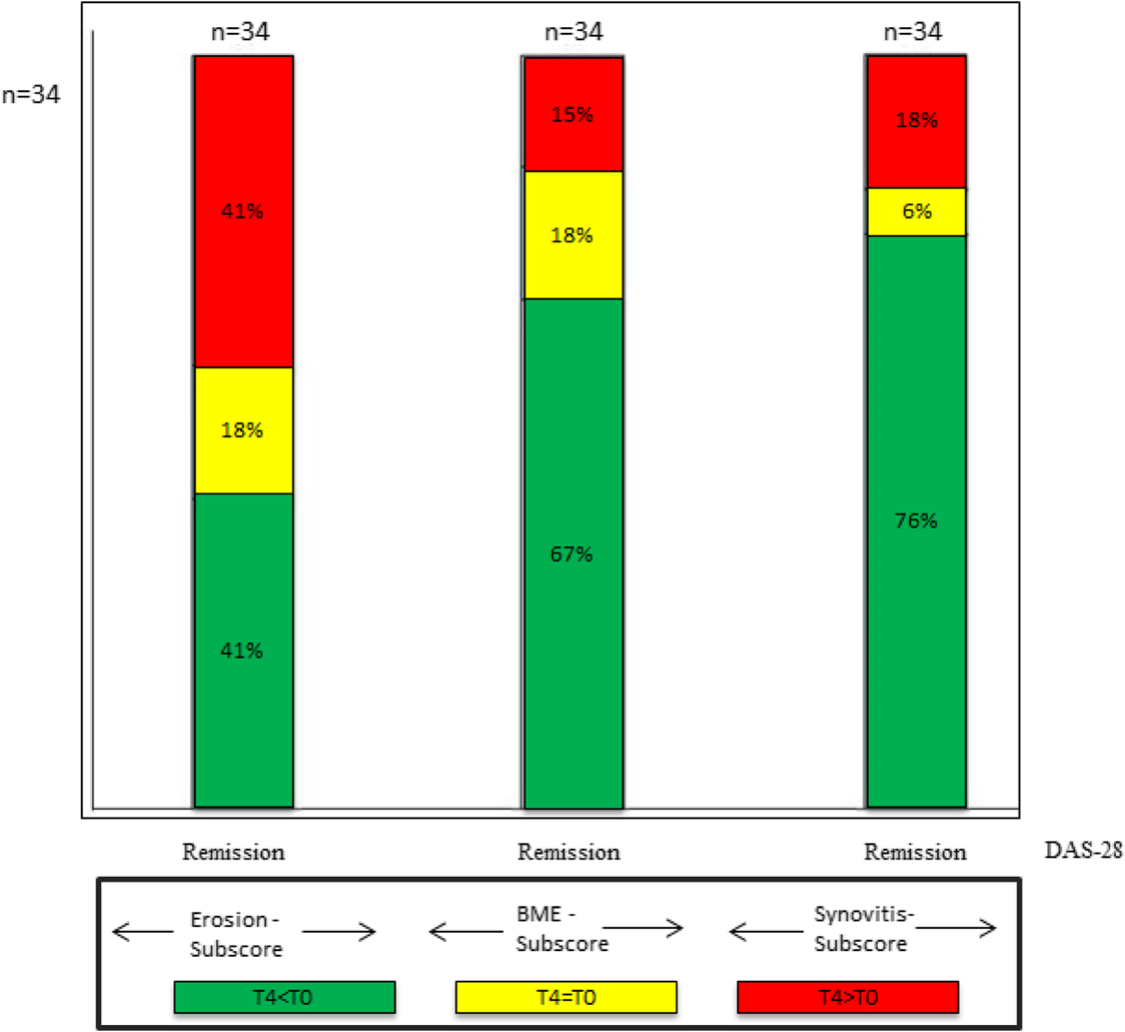
Comparison of patients who reached DAS28-remission to changes in Erosion-subscore, Bone-Marrow Edema (BME)-subscore and Synovitis-subscore of the RAMRIS Score. *Green coloured sections*: improvement in the RAMRIS-subscores; *yellow coloured sections*: equal values; *red coloured sections*: worsening in RAMRIS-subscores

Thirty four of the 71 patients who improved in DAS28 reached EULAR remission. Despite remission, 41% of all patients who attained remission showed an increased ERO-subscore after 12 months (T4) (Fig. 5).

### MRI changes with respect to EULAR response

**Fig. 6:**
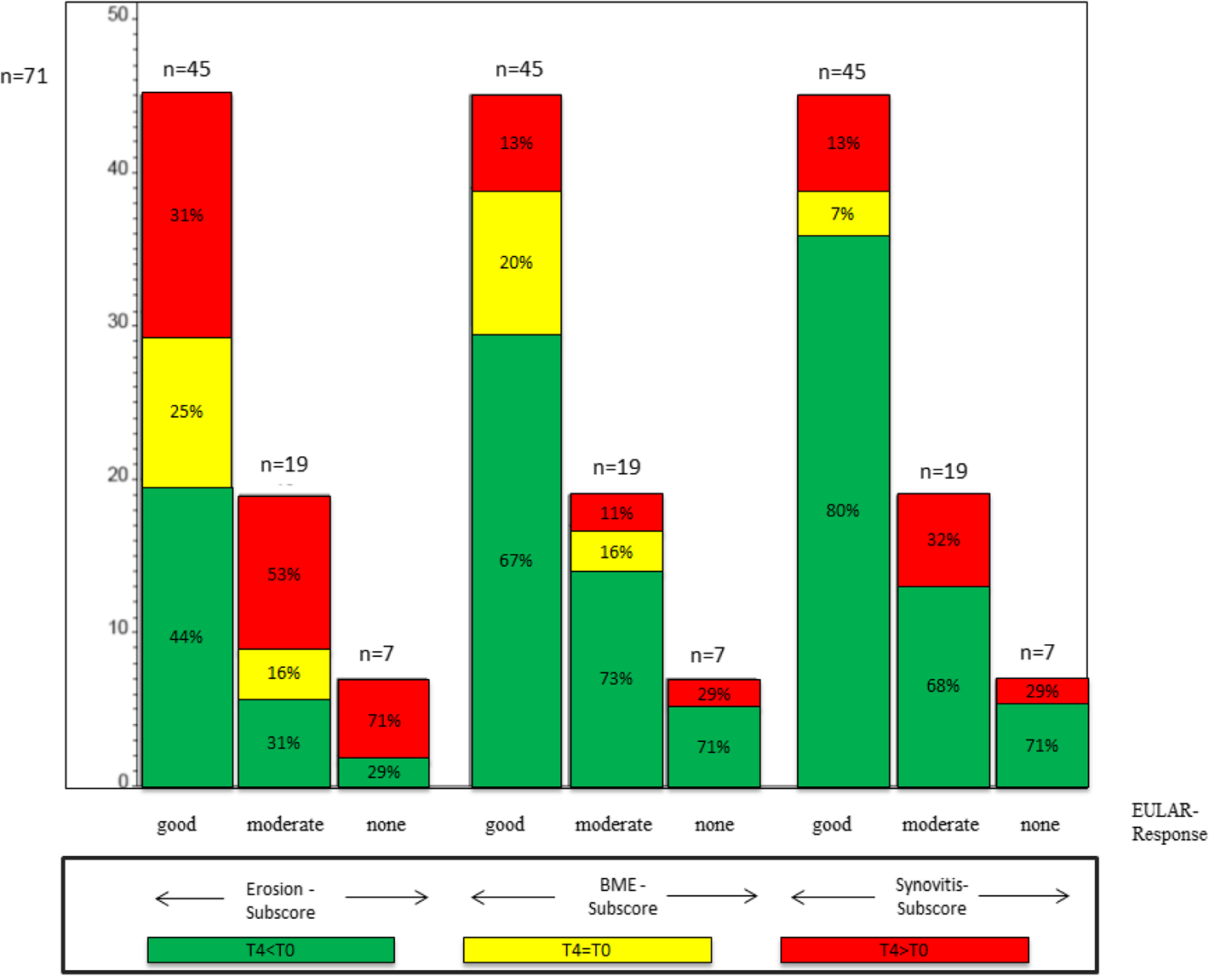
Comparison of patients who improved in DAS28 after 12 month to changes in Erosion-subscore, Bone-Marrow Edema (BME)-subscore and Synovitis-subscore of the RAMRIS Score. *Each left column*: patients who reached good EULAR-response regarding the EULAR response criteria (DAS28) after 12 month (T4 < T0); each middle column:patients moderate EULAR-response (T4 = T0); each *right column*: patients who reached none EULAR-response but improved in DAS28 after 12 month (T4 > T0). *Green coloured sections*: improvement in the RAMRIS-subscores (ERO, BME or SYN); *yellow coloured sections*: equal values; *red coloured sections*: worsening in RAMRIS-subscores

**Fig. 7:**
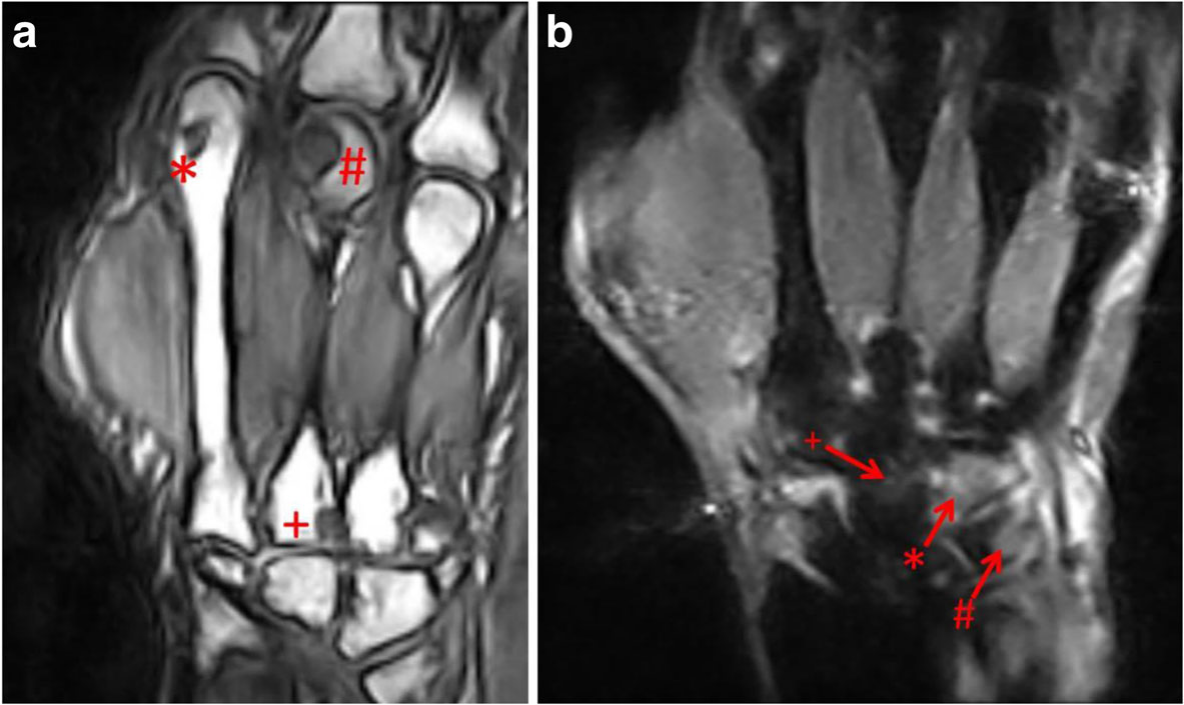
**a** Nativ T1-weighted image in coronal orientation. Erosion grade 1 in the head of metacarpus D2 (_*_), erosion grade 3 in the head of metacarpus 3 (#). Additionally, erosion of the basis of metacarpus 3 (+). The RAMRIS score of this patient was 48. **b** Short-Tau-Inversion-Recovery Sequenz (STIR) in coronal orientation: Osteoedema in Os hamatum (arrow with_*_) and Os triquetrum (*arrow with #*) grade 3. Additionally grade 2 osteodema in Os scaphoideum (*arrow with +*). The whole RAMRIS score was 43

Of the 71 patients who improved in DAS28 after 12 months, 7 showed EULAR non-response, 19 had moderate and 45 good EULAR responses. An increase of erosions was found in 71.4% of non-responders, 52.6% of moderate responders, and 31.1% of good responders at T4, all compared to baseline (Fig. 6). Representative MR-Images are shown in Fig. 7.

## Discussion

Remission is the ultimate goal in RA-therapy. This has been underscored by successful applications of the Treat-to-Target (T2T)-strategies in studies and clinical practice in the last few years [2]. Interestingly, MRI does not always reflect clinical improvement, but on the contrary, does show persisting or progressive joint pathologies in a considerable number of cases in most studies [25, 26]. However, the presence of erosions is associated with a high risk of progression of the disease, while this was only shown for erosions in conventional x-rays, yet [27, 28]. Up until now, therapy response criteria like the well-established EULAR response criteria are based on different constellations of clinical data, while matching MRI criteria are not available. In our study, a high number of 94% of patients showed erosions on MRI in at least one region. Importantly, roughly 40% of all patients who improved in DAS28 or who were in EULAR-defined remission, showed an increase of MR-detectable erosions after 12 months. Approximately 72% of these patients who worsened in ERO-subscore showed a subscore-deterioration of at least 2 points, so that an inaccuracy of the measurement is unlikely and a veritable increase of the MR-detectable erosivness must be assumed. Moreover, there was no relevant distinction between early and late RA, as even patients with a short disease-duration (less than 6 months) progressed.

The course of erosive changes depended on EULAR response in the current study: patients showing DAS28 improvement but EULAR non-response presented an increase of erosiveness in almost 72% of the patients, while only 31% of patients with good EULAR response had progressive erosions in MRI. Thus, our data is in accordance with a study by Van Gestel et al. who demonstrated that the improvement regarding the EULAR-response criteria is associated with less disease progression considering the clinical and conventional radiological course (highly sensitive imaging tools like MRI were not considered in this study) [8]. In contrast, it has been demonstrated that up to 20–30% of patients reaching clinical remission showed progressive erosive joint damage (silent progression) [9, 29]. Regarding the presented data, the proximal MCP-2 joints were most frequently affected by worsening in the ERO-subscore followed by the trapezoid bone and the proximal MCP-joints 3 and 4. The PIP joints were almost not affected (1/29). Regarding our additional analyses for possible predictive markers for silent progression (age, sex, antibody status, systemic inflammation (CRP) and RAMRIS-subscores), there were no statistical significant associations. However, we note that the study was not powered for specific subgroup analysis. It is known that erosive changes and BME detected by MRI lead to bone erosions which can be depict by conventional x-rays later on [16]. There is a lot of evidence that erosive progression in conventional x-rays is related to functional loss in the course of disease [30–32], while there is a lack of long term MRI data investigating the functional meaning of MR-detectable erosions, yet. Regarding that, long-term studies focused on this question are urgently needed.

Due to these issues, supplementary use of MRI scans could be of additional value to evaluate the therapy response, for example by using a smaller field of view to achieve a shorter examination time. In summary, MRI data in clinical routine confirm a high rate of silent progression despite DAS28 improvement or remission.

This study has some limitations. First, low-field MRI is used which is known to have a poorer local resolution in comparison to high-field MRI. Moreover, this multicentre study was a “real life” study without a static protocol, so that some patients were lost to follow-up or were excluded due to incomplete data. The study is not controlled for confounders such as RF, CCP-status, smoking or ethnicity. Moreover, we cannot completely exclude that progressive erosiveness detected by low-field MRI overestimates the risk of progression. In addition to that, it must be recognized that erosions were scored by MRI which is known as a very sensitive tool and could lead to occasionally over-interpretation. Hence, some sequences (for example STIR-sequences) are not fully comparable to high-field MR-scans due to the poorer resolution. To solve this last issue, both compound scores, such as the DAS28 and MRI based scores (e.g. RAMRIS), should be evaluated against gold standards such as functional or conventional radiological outcome measures in the long term in future studies.

## Conclusion

In conclusion, approx. 40% of patients demonstrated a progressive erosive RA detected by MRI despite DAS28 improvement or EULAR remission. Data is accumulating that DAS28 remission may be an insufficient therapy goal in RA. This is the first study showing the very high number of MRI-progression in RA patients despite clinical remission. Hence, MRI should be considered as a secondary outcome measure in interventional therapeutic trials with subsequent observational extension including functional measures and conventional x-rays to systematically assess this question.

## Abbreviations

ACR: American college of rheumatology
BME: Bone marrow edema
CI: Confidence interval
CRP: C-reactive protein
DAS28: Disease activity score 28
DMARD: Disease modifying anti rheumatic drug
ERA: Early rheumatoid arthritis
ERO: Erosion
ESR: Erythrocyte sedimentation rate
EULAR: European league against rheumatism
Gd-DTPA: Gadolinium-diethylenetriaminepentaacetic acid
MCP joint: Metacarpophalangeal joint
MRI: Magnetic resonance tomography
MTX: Methotrexate
OMERACT: Outcome Measures in RA Clinical Trials
RA: Rheumatoid arthritis
RAMRIS: Rheumatoid arthritis magnetic resonance imaging score
STIR: Short tau inversion recovery
SYN: Synovitis
